# An Update of Nucleic Acids Aptamers Theranostic Integration with CRISPR/Cas Technology

**DOI:** 10.3390/molecules27031114

**Published:** 2022-02-07

**Authors:** Mina Roueinfar, Hayley N. Templeton, Julietta A. Sheng, Ka Lok Hong

**Affiliations:** 1 Department of Biomedical Sciences, College of Veterinary Medicine and Biomedical Sciences, Colorado State University, Fort Collins, CO 80523, USA; mina.roueinfar@colostate.edu (M.R.); hayley.templeton@colostate.edu (H.N.T.); juliettasheng@gmail.com (J.A.S.); 2 Department of Pharmaceutical Sciences, Nesbitt School of Pharmacy, Wilkes University, 84 W. South Street, Wilkes-Barre, PA 18766, USA; 3 Department of Pharmaceutical Sciences, School of Pharmacy, Notre Dame of Maryland University, 4701 North Charles Street, Baltimore, MD 21210, USA

**Keywords:** CRISPR, aptamer, theranostic, biosensing, molecular recognition elements (MRE)

## Abstract

The clustered regularly interspaced short palindromic repeat (CRISPR)/Cas system is best known for its role in genomic editing. It has also demonstrated great potential in nucleic acid biosensing. However, the specificity limitation in CRISPR/Cas has created a hurdle for its advancement. More recently, nucleic acid aptamers known for their high affinity and specificity properties for their targets have been integrated into CRISPR/Cas systems. This review article gives a brief overview of the aptamer and CRISPR/Cas technology and provides an updated summary and discussion on how the two distinctive nucleic acid technologies are being integrated into modern diagnostic and therapeutic applications

## 1. Introduction

The clinical advancement of the messenger RNA (mRNA) vaccine to combat the COVID-19 pandemic has been a hallmark of success in functional nucleic acids. In addition to mRNA, other functional nucleic acids, such as aptamer, have also gained increasing attention in biomedical and biotechnology research. Aptamers as the targeting agents for physiologically important targets have been heavily researched since aptamer discovery in 1990. Furthermore, one of the most novel applications is its integration to enhance the utility and precision of the CRISPR/Cas (clustered regularly interspaced short palindromic repeats/cellular apoptosis susceptibility) system for functions beyond genomic editing. This review is written to provide a snapshot of the recent innovative research regarding aptamer and CRISPR/Cas system.

### 1.1. Overview of Aptamers

The discovery of aptamers began in the early 1990s in two different laboratories (Larry Gold and Jack William Szostak). They independently established the in vitro selection and amplification process to identify the desired nucleic acid sequences (DNA or RNA) capable of binding to their specific targets [1,2,3]. Szostak’s lab gave for these binding elements the term aptamer [2]. Aptamers are short nucleic acid molecules that are artificially selected against their desired targets. Their ability to bind to the desired target is determined by folding into a tertiary structure and their orientations in solution or immobilized on solid platforms [4,5]. Aptamers can recognize a wide range of molecules, such as proteins, enzymes, toxins, or ions, and bind to these specific targets with high affinity and specificity. Aptamers have also been investigated as novel tools in targeted therapeutics, disease diagnostics, and imaging applications [6,7,8]. Furthermore, aptamers have a number of advantages over antibodies, such as higher thermal stability, ease of synthesis, no or minimum immunogenicity, high flexibility, and ease of modifying. These features contribute to the engineering of these molecules into sensors [9].

**Figure 1:**
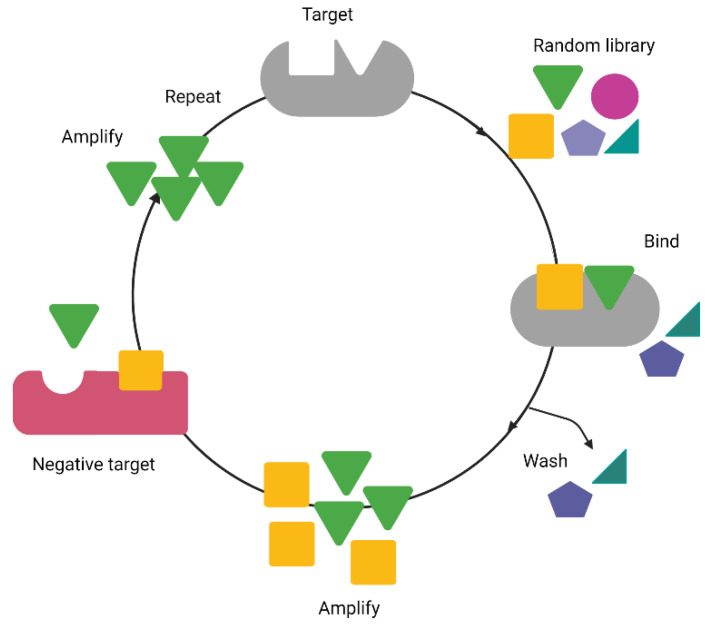
An illustration of the basic schematic of the Systematic Evolution of Ligands by Exponential Enrichment (SELEX) process. The random library of oligonucleotides is subjected to alternate incubation cycles with the target of interest and counter (negative) targets. The process is typically repeated up to 15 times to increase the binding affinity and specificity of the oligonucleotide library. The final enriched library may contain one or more candidate aptamers. The figure was created with BioRender.com.

The diverse molecular binding profile of aptamers has been successfully employed to screen different biomarkers frequently associated with diseases [10]. Aptamers are generally developed through an in vitro selection process from an extensive random oligonucleotide library called Systematic Evolution of Ligands by exponential enrichment (SELEX) [3]. A typical SELEX process usually starts with incubation of the large random oligonucleotide library with a target molecule, followed by the separation of bound and unbound sequences to the specific target and amplification of the bound sequences by PCR. This process is repeated until the pool of high affinity aptamers is sufficiently enriched. Counter-SELEX or negative rounds of SELEX typically follow the positive selection rounds to prevent non-specific bindings to pre-determined undesired targets [11] (Figure 1).

The original SELEX methodology has limited efficiency due to the insufficient partitioning of bound and unbound sequences. Typically, several rounds of SELEX (typically 5 to 15 rounds) are required to increase the enrichment of the library and stringency of the resulting aptamer [12]. It is now known that the efficiency of traditional SELEX is low due to limited partition ability, insufficient stability, and low specificity [13]. Therefore, different variants of SELEX, such as cell-SELEX [14], capture-SELEX [15], capillary electrophoresis SELEX [16], in vivo SELEX [17], and graphene oxide SELEX (GO-SELEX) [18], were developed to improve and diversify the aptamer selection efficiency.

For instance, cell-SELEX was developed to screen for aptamers that can bind to cell surface markers in healthy and diseased live whole cells. Aptamers identified from cell-SELEX can be used as the targeting agent to deliver a payload for cancer diagnosis and treatment [19]. Additionally, aptamers can detect functional cell surface markers involved with post-translational modification of enzymes and cofactors that influence their function [20]. Further, cell-SELEX can be utilized to identify unknown biomarkers with unknown roles and functions in pathogenesis. For example, Xu et al. reported a panel of aptamers that can bind to possible common proteins expressed in lung adenocarcinoma, ovarian cancer, and embryonic kidney cells while maintaining specificity against leukemia or cervical cancer [21].

Multiple articles have thoroughly reviewed the theranostic application of aptamers as a targeting agent for cancer biomarkers [6,22,23]. Furthermore, beyond the mentioned aptamer applications, there has been increasing attention for integrating the CRISPR/Cas genetic editing technique.

### 1.2. Overview of the CRISPR/Cas System

Clustered regularly interspaced short palindromic repeats (CRISPR) were first identified in bacteria and archaea as a defense mechanism to attack bacteriophage [24]. Briefly, a phage infects the prokaryote cell and uses the host machinery to make phage proteins for replication. When the phage initially attacks, CRISPR recognizes the DNA mismatch and creates cellular apoptosis susceptibility (CAS) proteins that subsequently take and cut the DNA apart. The prokaryote cell also catalogs the DNA into the CRISPR system to prevent damage from future attacks [25]. Among the three CRISPR/Cas systems that were identified in bacteria, the type II (CRISPR/Cas9) system in *Streptococcus pyogenesis* is widely used for genome editing in mammalian cells [26]. The CRISPR system contains two main components: the guide RNA is complementary to specific target DNA loci, and the nuclease works as a pair of molecular scissors to cut the target DNA sequence. Cas9 is the most commonly used nuclease in CRISPR genome engineering experiments, which can cleave the DNA at specific loci in the presence of crisprRNA (crRNA) and tracrRNA complex, which are referred to as guide RNA (gRNA) [27].

**Figure 2:**
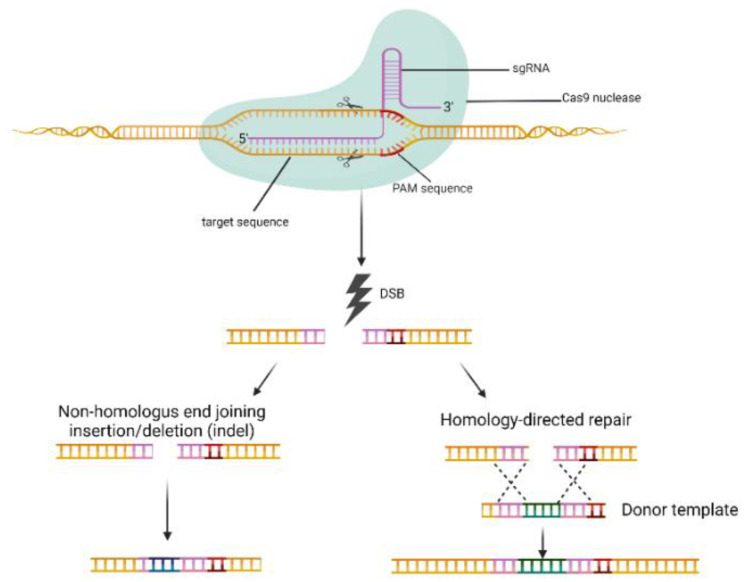
CRISPR/Cas9 genome editing system. Cas9 induced double-strand breaks (DSBs) at the target DNA loci. The two major DNA repair mechanisms are divided into two pathways in mammalian cells: (1) non-homologous end joining (NHEJ), and (2) homology-directed repair (HDR). The NHEJ pathway induces precise insertions or deletions (indels). The HDR mechanism uses donor DNA templates often from sister chromatids or an exogenous DNA template to generate knock-ins and base substitutions between DSB sites. The figure was created with BioRender.com.

CRISPR/Cas9 can be used to precisely edit gene sequences through the use of a 20-nucleotide guide RNA (gRNA), which targets complementary DNA sequences a few base pairs upstream of the protospacer adjacent motif (PAM) sequence [28]. Cas9 nuclease then recognizes the PAM sequence and induces a double-strand break (DSB) at the target loci [29]. The double-strand breaks can be repaired through either non-homologous end joining (NHEJ) or homology-directed repair (HDR) in mammalian cells. The repair mechanisms can induce gene editing at the repair site by inserting a new DNA sequence into the existing sequence, which can lead to frame shifts in an open reading frame or knockout the gene [30] (Figure 2).

Over the past few years, several Cas proteins were discovered with different functions. For example, both Cas 9 and 12 share a very similar structure, but Cas9 generates blunt-ended DNA breaks close to the PAM site, while Cas 12 induces DNA breaks far from the PAM recognition site [31]. In addition, while some nucleases cleave DNA, other nucleases targeting mRNA in mammalian cells, such as Cas13a and Cas13b, provide alternative avenues in genome editing [32].

Traditionally, there are several ways to perform genetic engineering, but most are cumbersome. These techniques include small interfering RNA (siRNA), zinc finger nuclease (ZFN), and transcription activator-like effector nucleases (TALEN). siRNAs are derived from double-stranded RNAs (dsRNAs) and mediate the cleavage of target RNAs [33]. In the cells, siRNAs incorporate into other proteins from the Argonaute (AGO) protein family to form an RNA-induced silencing complex (RISC) [34]. siRNAs possess some disadvantages, including the lack of an appropriate and high efficiency in vivo delivery system to protect siRNAs against endonucleases or exonucleases. This can potentially reduce the target accessibility and specificity of this technique [35].

Furthermore, ZFNs are composed of a zinc finger protein domain that is coupled with a site-specific nuclease for cutting DNA. The cutting is highly site specific and is overall very complex. ZFNs are also associated with the high cost of protein domain construction and inaccurate cleavage. The third tool, TALENs, relies on their pathogenic origin’s ability to secrete transcription activator-like effectors to their host cell’s cytoplasm. They can also bind to DNA and suppress target genes by mimicking eukaryotic transcription factors [36]. However, they are still relatively costly to use and are tricky to design.

The discovery of the CRISPR/Cas9 system has revolutionized the field of molecular genetics and genome editing. One of the most promising applications of the CRISPR/Cas9 genome editing tool is to treat human genetic diseases by editing the human genome. It also facilitates the process of creating animal models to study human diseases [37,38]. CRISPR/Cas9 has rapidly proved itself to be the most popular and powerful genome-editing tool because it is relatively easy to use and cost efficient. In addition, it offers direct targeting by only modifying the 20-nucleotide target sequence of the gRNA, which makes it more efficient than the ZFNs and TALENs genome engineering systems [39]. Hence, CRISPR/Cas9 technology has been used worldwide to accelerate biological research in many areas, such as cancer and gene therapy. Moreover, CRISPR has many potential applications, such as improving agriculture, environmental engineering, plant engineering, and epigenetics [40].

### 1.3. CRISPR/Cas System Limitation

Studies show that genome editing can prevent and treat disease by targeting the disease-specific gene. Therefore, the safety and side effects of genome-editing procedures are critical and considered a limiting factor. Although the CRISPR/Cas9 system facilitates genome editing efficiency and offers many promising benefits for human health, there are serious concerns about the biosafety and technical aspects related to the development of the CRISPR/Cas9 system [41]. One of the significant challenges for researchers conducting CRISPR experiments is preventing unwanted genome modifications, known as off-target effects [42]. The target efficiency and specificity of CRISPR/Cas9 are defined by the 20-nucleotide sequence of gRNA and PAM sites downstream of the target loci. The potential off-target cleavage happens when there are more than three base pair mismatches between the target sequence and the 20 nucleotides of gRNA [43]. Additionally, studies showed that four mismatches in the PAM-distal part could generate off-target effects [44]. Off-target effects can change the function of normal genes and induce large deletions and genomic rearrangement, which cause genome instability and raise the unknown health risk in clinical applications [43,45].

Another limitation of CRISPR/Cas9 is the lack of in vivo targeted delivery systems. The successful delivery of the CRISPR/Cas9 system into the nucleolus of the target cells is essential for biomedical and clinical applications, such as gene therapy. Researchers are utilizing different vehicles to deliver the CRISPR/Cas9 system. These vehicles exist in non-viral, viral, and ribonuclear complexes form factors [46]. Viral vectors can exist in the forms of adenoviral, adeno associated virus (AAV), and lentiviral vectors. These vectors deliver the Cas9 system-encoding cassettes. Packaging in adeno-associated virus vectors is the current most commonly used method of delivery [47,48,49]. While AAVs offer high efficiency in gene delivery and expression, the potential of carcinogenesis, immunogenicity, and large delivery number of viruses (shelled) to the body are of great concern. Moreover, there are also difficulties in large-scale vector production [50,51].

Thus, it is worth considering another delivery system such as aptamer with high efficiency and specificity toward the target cells and tissues. Aptamer’s long history of experimental use for in vivo targeted delivery of therapeutic agents, favorable binding profile, and characteristics would allow direct modification to improve the Cas9 system delivery efficacy and limit errors.

## 2. Aptamer Integration in CRISPR/Cas System

**Figure 3:**
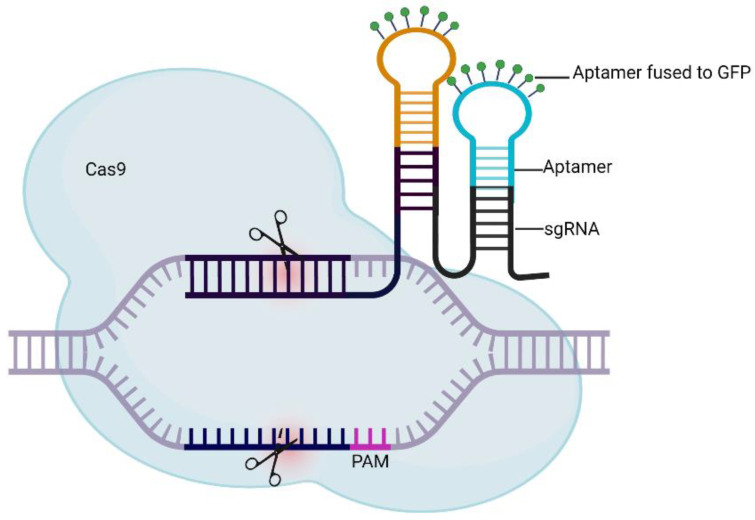
Schematic illustration of aptamer integration in CRISPR/Cas system. The aptamer construct is integrated into the sgRNA scaffold and can be detected with a green fluorescent protein, which is fused to aptamer stem–loop structure. Figure is adapted from Khosravi et al. [52]. The figure was created with BioRender.com.

The following section reviews recent (2016 to 2021) reports that have integrated aptamers into the CRISPR/Cas system for (1) improving CRISPR specificity, (2) application in cancer theranostics, (3) imaging and biosensors, and (4) application in infectious disease, with highlights on the development of aptamer/CRISPR detection assays for severe acute respiratory syndrome coronavirus 2 (SARS-CoV-2). A table summary is provided at the end of this section (Table 1, Figure 3).

### 2.1. Improvement of CRISPR Specificity

A method of delivery that may alleviate potential off-target effects seen with CRISPR is through aptamer-mediated delivery of the CRISPR/Cas system. One such way for aptamer control of CRISPR can be achieved by regulating CRISPR single guide RNA (sgRNA). Kundert et al. explored the possibility of sgRNA control via aptamers to generate ligand-responsive sgRNAs. The authors developed ligand-responsive sgRNAs by using theophylline binding RNA aptamers. This approach allowed the sgRNAs to activate and deactivate the CRISPR/Cas9 action using theophylline. The theophylline action was also dose dependent. In addition, the authors also integrated a second aptamer specific for 3-methylxanthine to gain independent control of multiple genes with two ligands [53].

Lin et al. developed a similar strategy using small molecule-activated allosteric aptamer regulating (SMART)-sgRNAs [54]. Furthermore, by integrating the theophylline binding RNA aptamer and theophylline into protein-unrecognized regions of sgRNA, the authors also achieved an increased temporal control of the CRISPR/Cas9 system. The above methods have the potential to enable temporal control of several genes aiding in the specificity and safety of genomic editing.

Another method that could decrease the off-target effects of CRISPR is to block persistent Cas9 activity in cells. Zhao et al. utilized in vitro selection to generate an inhibitory DNA aptamer against Cas9. This inhibitory aptamer could bind to Cas9 in low nanomolar affinity [55]. This high-affinity binding interaction lowered the Cas-9 directed genome editing within cells and reduced off-target effects. As previously discussed, CRISPR relies on the generation of DNA double-stranded breaks (DSB), leading to genotoxicity. Collantes et al. generated an RNA aptamer-mediated base editing (BE) system called Pin-point^TM^ to limit the need for DSB generation. In this system, the guide RNA (gRNA) was engineered to contain an RNA aptamer that recruits different DNA deaminases to enable the introduction of point mutations to DNA or RNA without the formation of DSBs. As a result, the Pin-point^TM^ system demonstrated lower off-target editing than previous base editing models [56].

Typically, CRISPR-Cas9 is delivered into cells via a plasmid vector, triggering an innate immune response (IIR) inside the cells. Subsequently, it can lead to ineffective gene editing and transgene expression. Zhan et al. overcame this challenge by developing artificial nucleic acid molecules (ANAMs) composed of RNA aptamers that bind to β-catenin and NF-κβ, which play a significant role in developing IIR immune response [57]. It was found that the ANAMs were able to inhibit IIR, thereby improving gene editing and transgene expression of the CRISPR-Cas9 system.

Overall, the combination of aptamer and CRISPR technologies provides a system with decreased off-target effects and increased temporal control of gene editing offering a more efficient and safe method of genomic editing.

### 2.2. Application in Cancer

Even though the CRISPR/Cas9 system is a versatile genome-editing tool, it lacks cell-specific in vivo tumor-targeted delivery systems. Aptamers offer a promising solution to this problem by providing high sensitivity and specificity, low immunogenicity, and small size.

Several studies have shown the potential of aptamer-mediated CRISPR/Cas9 systems in cancer therapeutics. Liang et al. developed an aptamer-functionalized lipopolymer to deliver CRISPR/Cas9 to edit vascular endothelial growth factor (VEGFA) in osteosarcoma [58]. The authors used an osteosarcoma cell-specific aptamer LC09 to generate a LC09-functionalized PEG-PEI-Cholesterol (PPC) lipopolymer capable of capturing CRISPR/Cas9 plasmids that encoded for VEGFA gRNA and Cas9. This system led to the selective delivery of CRISPR/Cas9 to osteosarcoma tumor cells providing effective VEGFA genomic editing. As a result, it decreased VEGFA expression and ultimately led to a decrease in osteosarcoma malignancy and lung metastasis [58].

Zhen et al. used an aptamer-cationic liposome to modify CRISPR gRNA. Cationic liposomes were linked to an RNA aptamer bound specifically to prostate cancer cells [59]. These liposomes were used to deliver CRISPR/Cas9 to prostate tumor cells where it targeted polo-like kinase 1, a survival gene. This method resulted in highly specific cell binding and significantly decreased polo-like kinase 1 expression in vitro. A high regression of prostate cancer in vivo was also reported in their study [59].

Tumor-derived extracellular vesicle (TEV) proteins are potential diagnostic markers for cancer. Xing et al. developed an assay that allowed for the detection of TEV proteins with high sensitivity, known as the apta-HCR-CRISPR assay [60]. This assay utilizes a dual application of hybridization chain reaction (HCR) to amplify a TEV aptamer which was further amplified by CRISPR-Cas12a activities providing a valuable method for TEV proteins detection [60].

The TEV proteins, CD109, and epidermal growth factor receptor (EGFR) are present in nasopharyngeal carcinoma (NPC), and all markers decrease significantly after radiotherapy, suggesting their role in NPC diagnosis. Li et al. developed an aptamer-CRISPR/Cas12a assay that enabled the detection of CD109 and EGFR at as low as 100 particles/mL [61]. This assay quickly and specifically determined levels of CD109 and EGFR to aid in the detection and diagnosis of NPC [61]. These examples showed that aptamer-mediated CRISPR/Cas systems might offer a universal method of cell-specific CRISPR/Cas delivery, thus enhancing the theranostic value of the CRISPR/Cas system.

### 2.3. Application in Imagery and Biosensors

In addition to enhancing theranostic applications, combining CRISPR technology with aptamers also showed values in imagery and biosensor applications. For instance, CRISPR could enhance chromatin imagining techniques and better understand the physiology of development and vital biological processes [52,62].

Wang et al. improved an existing Cas9 CRISPR/Cas9 live imaging system in *Streptococcus pyogenes* by using a sgRNA scaffold fused to MS2 or PP7 RNA aptamers [62]. The addition of RNA aptamers enabled two-color CRISPR labeling with an improved signal-to-background ratio of chromatin imaging, providing overall enhanced quality of telomere labeling.

The colorimetric analysis is a common tool to quantify the concentration of a specific compound in a sample with the addition of a color-changing reagent. Recently, colorimetric methods have gained popularity due to their lack of requirement for highly sophisticated equipment. However, the development of colorimetric sensing with reliable sensitivity has been somewhat limited. Abnous et al. developed an ultrasensitive colorimetric aptasensor by using the catalytic activity of gold nanoparticles (AuNPs) and CRISPR-Cas12 with rolling circle amplification (RCA) [63]. The aptasensor was reported to detect varying concentrations of carcinogenic toxin, aflatoxin M1 (AFM1), in spiked milk at a concentration as low as 0.05 ng/L. In the presence of phi29 DNA polymerase and T4 DNA ligase, CRISPR-Cas12a with RCA became inactive with AFM1, forming large single-stranded DNA structures on the surface of AuNPs. After adding 4-nitrophenol, the digestion of the primer by CRISPR-Cas12a facilitated the color transition from yellow to colorless. Moreover, large single-stranded DNA structures were not observed on the surface of AuNPs. The result suggested that this colorimetric approach was ultrasensitive in the presence of AFM1 and provided a potential method to detect other harmful mycotoxins in similar real samples [63].

Another utilization of the CRISPR-Cas biosensor system is the detection of nucleic acid targets. Nucleic acid detection with CRISPR-mediated systems can provide inexpensive, selective, and rapid detection of many point-of-care pathogens and associated disease. CRISPR-Cas13a, combined with isothermal amplification, rapidly detected RNA or DNA at a single-base mismatch selectivity and attomolar sensitivity [64]. It was termed as specific high-sensitivity-enzymatic reporter unlocking (SHERLOCK). The authors recognized pathogenic bacteria, mutations in noncarcinogenic DNA and subsequently identified viral strains, such as the Zika and Dengue virus.

Additional studies demonstrated that CRISPR reporting could distinguish single-nucleotide variations (SNVs) in mitochondrial DNA. CRISPR-Cas9-mediated ligation was used to image SNVs in the ND4 and ND5 genes at a single-molecule resolution [65]. Using this technique, the authors observed the transfer of mtDNA between cells, which was thought to increase the risk for certain age-associated diseases. These data suggest CRISPR-mediated detection of SNVs in single cells in the genome could be a useful tool in understanding the role of SNVs in disease and genetic diagnoses [65].

Rosch et al. further generated highly specific aptamers that detect native membrane proteins using the CRISPR-mediated SELEX [66]. Many in vitro compounds that bind to native membrane proteins can also unintentionally bind to non-target membrane proteins. The authors attempted to address this issue with isogenic pairs of cells. Following the knockout of solute carrier family 2 member 1 (SLC2A1), a GLUT1 glucose transport transcription factor in epithelial cells, cell-SELEX lines were generated against knockout and wild-type cells. After several rounds of cell-SELEX, it generated highly specific binding reagents to GLUT1. The result suggested a CRISPR-mediated SELEX approach involving ultra-specific aptamers could generate reagents with selective affinity for native membrane proteins [66].

On its own, the CRISPR/Cas system has been limited clinically due to its low versatility and moderate sensitivity for non-nucleic acid targets. Li et al. developed an immunoassay using aptamer-linked CRISPR/Cas12a [67]. In this assay, an aptamer sequence was added to the 5′ end of the activator DNA of Cas12a. Through RNA hybridization of the aptamer flanked activator DNA and the CRISPR/Cas12a complex, molecule recognition, and signal generation occur similar to an ELISA assay. This enables the detection of biomarkers at an ultrasensitive level and at a low cost, making it ideal for bioassay and medical diagnostics applications [67].

Nui et al. developed a versatile biosensor method to identify small molecules known as molecular radar (Random Molecular Aptamer-Dependent CRISPR-Assist Reporter), utilizing a CRISPR-Cas12a reporter system and a single-stranded DNA aptamer specific for the target [68]. The target in this study was adenosine-5′-triphosphate (ATP) and was detected with incredibly high sensitivity and specificity utilizing the CRISPR-Cas12a aptamer system. Peng et al. also used the CRISPR-Cas12a system with an ATP aptamer to obtain specific detection of ATP in 40 min, illustrating the ease and potential of CRISPR and aptamer-based methods for detecting small molecules [69].

Furthermore, fluorescent and electrochemical biosensors were also developed with DNA aptamer integration with the CRISPR-Cas12a reporter system. Xiong et al. developed a CRISPR-based ATP sensor using the structural switching ATP DNA aptamer. The authors reported detection of ATP quantitatively under room temperature in less than 15 min [70]. Dai et al. developed an electrochemical-based CRISPR sensor that integrated the DNA aptamer to detect transforming growth factor 1 (TGF-1) protein in clinical samples [71]. Deng et al. developed a sandwich immunosenor using antibodies and DNA aptamer that bind to interferon-gamma [72]. The fluorescence signal was amplified by the CRISPR/Cas12a system. The immunosensor was able to detect the target in complex biological samples, including serum, whole blood, sweat, and saliva. Lu et al. combined upconversion nanoparticles, luminescent resonance energy transfer, and dual-aptamer regulated CRISPR/12a system to detection cardiac troponin I level in virally infected mice [73]. The luminescent signal was boosted by a three-dimensional photonic crystal boated biochip to achieve a limit of detection at 7.6 pg/mL. The reported sensitivity was approximately 13 times better than commercial enzyme-linked immunoassay kits.

### 2.4. Application in Infectious Diseases

The SARS-CoV-2 novel coronavirus has led to millions of deaths since the World Health Organization declared the COVID-19 pandemic in March 2020. Nucleic acid (NA)-based tests are widely used to detect RNA viruses, such as SARS-CoV-2. CRISPR approaches allow for increased sensitivity and specificity in NA-based tests.

Wang et al. reported a transcription amplification system using a light-up RNA aptamer signaling-CRISPR Cas13, which can detect as low as 82 copies of SARS-CoV-2 [74]. This system also allowed the highly pathogenic SARS-CoV-2 variant, D614G, to be differentiated, aiding in diagnosing and monitoring SARS-CoV-2. Being able to diagnose SARS-CoV-2 rapidly and accurately at a low cost has an important role in the treatment and prevention of the virus. Zhao et al. combined CRISPR/Cas12a and two DNA aptamers to create an ultrasensitive PCR-free antigen detection platform to detect the SARS-CoV-2 virus [75]. The platform detected the specific nucleocapsid protein at the single virus level or about two copies per microliter in the saliva or serum sample. The entire detection process took only 20 min. Han et al. developed a CRISPR/Cas12a electrochemical aptasensor to detect the SARS-CoV-2 viral nucleocapsid protein [76]. Their system achieved a detection limit of 16.5 pg/mL within 30 min of sample treatment. It also demonstrated target detection in milk, tap water, and serum samples. CRISPR and aptamer technology integration showed great promise to provide solutions aiding the fight against SARS-CoV-2.

The COVID-19 pandemic has set a current example that rapid and specific diagnosis and treatment of infectious diseases are critical. Xu et al. reported a CRISPR-Cas12a assisted rolling circle amplification (RCA) and dual aptamer technique for bacteria detection, specifically methicillin-resistant *Staphylococcus aureus* (MRSA) [77]. The dual aptamers recognize MRSA-specific proteins on the surface of bacteria, while CRISPR-Cas12a aids in trans-cleavage, permitting dual amplification of the MRSA nucleic acid signal. This novel method provides a highly sensitive and accurate method for MRSA detection, offering promising applications of aptamer and CRISPR-based bacteria detection [77].

The ability to identify trace amounts of bacteria would further aid in the early detection and treatment of infectious diseases. Penicillin-binding proteins 2a (PBP2a) are found on the surface of MRSA. Wei developed a method for the detection of MRSA that allows for trace amounts of the *Staphylococcus aureus* (SA) bacteria to be detected through the use of an allosteric probe is used to identify SA via a PBP2a-specific aptamer [78].

Zhang et al. reported a method to detect pathogenic bacteria using a light-up RNA aptamer signaling-CRISPR-Cas13a system for biosafety control via the detection of viable pathogens [79]. *Bacillus cereus* can cause food poisoning, and quantification of viable bacteria can aid in the estimation of food spoilage to prevent food poisoning. Using the light-up RNA aptamer, Broccoli, live *Bacillus cereus* bacteria were accurately detected and quantified without using a chemical label as seen in other CRISPR-based labeling systems.

Liu et al. developed a hybridization chain reaction (HCR) based CRISPR-Cas12a electrochemical biosensor using the DNA aptamer that binds to *Salmonella typhimurium* [80]. The aptamer was released in the presence of the target bacteria, and then Cas12a cut the double-stranded DNA of HCR to release the electrochemical probe. The author reported detection limits of 20 CFU/mL in bacteria-spiked milk samples.

Overall, the combination of aptamer and CRISPR technologies has been used as a powerful tool for the development of screening assays, gene therapy, cancer treatment, imaging techniques, pathogenic bacteria detection/quantification, and SARS-CoV-2 diagnosis and treatment.

**Table 1:** Summary of aptamer–CRISPR areas of application.

Specific Application	Aptamer Type	CRISPR/Cas System	Pros	Cons	Types of Application	Reference
**Improvement of CRISPR specificity and off-target effects**	DNA/RNA	Cas9	Dose-dependent activation/inactivation On-target sgRNA allows for high regulation of multiple genes Inhibitory aptamers can bind Cas9 at low affinities	Control only under specific conditions Regulation of genome editing in reverse Potential risk of immunogenicity and toxicity	Genomic editing/therapeutic/diagnostics/research	[53,54,55,56,57]
**Osteosarcoma**	DNA	Cas9	Inhibits osteosarcoma and lung metastasis effectively Reduce VEGFA expression/secretion Decreased angiogenesis	Studies in mice do not completely translate to humans Use of lipopolymers only in phase II trials	Therapeutic	[58]
**Prostate cancer**	RNA	Cas9	Highly flexible for liposome modifications to target a myriad of diseases Highly selective for prostate cancer cells (specifically polo-like kinase 1) in vitro No significant toxicity Safer than cationic liposome	Requires the appropriate modification for highest efficacy	Therapeutic	[59]
**Tumor-derived extracellular vesicle protein**	DNA	Cas12a	Simple and easy to operate High affinity for surface protein targets on TEVs Clinically feasible and cost-effective	Kinetic efficiency is low Requires optimal conditions (i.e., hairpins with large loops, specific heating temperatures/durations)	Diagnostics	[60]
**Nasopharyngeal carcinoma**	DNA	Cas12a	Sensitive, specific detection of surface proteins Detects CD109+ and EGFR+ TEVs, good biomarkers for NPC diagnosis Small batch variation Stable and simple	Can only do one marker per run, lowers the accuracy of detection Low ability to determine specific amounts of each target protein Uses PCR, which requires cooling/heating cycles. Therefore, heat-stable DNA is required. Cost of RPA increases detection cost	Diagnostics	[61]
**Chromatin Imaging**	RNA	Cas9	Specific two-color labeling of repeating sequences Signal-to-background ratio enhanced in chromatin imaging	Not used on non-repetitive sequences yet Involves an extra protein construct compared to other methods Cell-line construction is more complex than other models	Imaging, Research	[62]
**Mycotoxin Detection**	DNA	Cas12a	Does not require sophisticated equipment Detection limit at 0.05 ng/L Highly selective for AFM1 Synthesis of old nanoparticles is convenient	Only tested in milk samples	Biosafety	[63]
**Native membrane protein detection**	DNA	Cas9	SELEX uses isogenic cell lines to specifically bind GLUT1 transporter Can be used to create reagents that bind a variety of specific cell membrane proteins in native conformation	Did not examine off-target CRISPR edits The efficiency of selection reduced by unintended effects of the SELEX procedure used SELEX process might have induced unintended mutations in cells Homologous family members not removed, reducing the hit rate of specific aptamers	Therapeutic, Diagnostic	[66]
**Immunoassay**	DNA	Cas12a	Direct relationship between non-nucleic acids and CRISPR-Cas12a system examined Can be directly used in enzyme-linked immunosorbent assay Simple for biosensing High detection for small molecules and tumor cells Nanoprobes easy to manufacture	The efficiency of the detection platform still needs to be improved	Diagnostics	[68]
**Small Molecule Bioassay (ATP)**	DNA	Cas12a	ATP detect method has high sensitivity and selectivity Novel characteristics of CRISPR-Cas12a described Novel fluorescent biosensor used fDNA-regulated CRISPR useful in point-of-care and field tests	Requires costly fluorescent reader for biosensors ATP biosensors are limited by oligonucleotide design The portable fluorimeter-based method shows reduced analytical performance	Diagnostics	[68,69,70]
**Transforming Growth Factor 1 (TGF-1)**	DNA	Cas12a	Rapid, cost-effective E-CRISPR highly specific for small viral nucleic acids Able to accurately quantify TGF-1	Steric hindrance effect of Cas endonuclease limited activity of trans-cleavage High concentrations reduced activity of Cas12a, leading to decreased diffusion	Diagnostics	[71]
**Interferon-gamma**	DNA	Cas12a	Sensitive detection of the target in complex biological samples	Special equipment is needed for fluorescent signal detection	Diagnostics	[72]
**Viral myocarditis–Cardiac Troponin I**	DNA	Cas12a	Sensitive detection of the target in mouse serum samples	Complex setup using upconversion nanoparticles and 3D photonic crystal	Diagnostics	[73]
**SARS-CoV-2 Detection**	RNA DNA DNA	Cas13 Cas12a Cas12a	Sensitive and rapid detection of pathogenic SARS-CoV-2 variants Detection of low viral titer	Diagnose active infection only	Diagnostics	[75] [75] [76]
**Methicillin-resistant *Staphylococcus aureus* (MRSA) Detection**	DNA DNA	Cas12a	Accurate and sensitive bacterial detection Decreased cost enabling worldwide use	Components of clinical samples may impair aptamer target binding	Diagnostics	[77] [78]
***Bacillus cereus* Detection**	RNA	Cas13a	Ability to detect live pathogenic bacteria Accurate estimation of food spoilage	Limited to *Bacillus cereus* detection	Biosafety	[79]
***Salmonella typhimurium* Detection**	DNA	Cas12a	Highly sensitive detection of live bacteria in milk samples	Highly dependent on fine tuning the aptamer concentration on sensor surface	Food safety	[80]

## 3. Conclusions and Future Perspective

CRISPR/Cas technology has revolutionized how scientists edit genes. Yet, it is not without limitations. Off-target effect and barriers of in vivo delivery are the early identified examples. For instance, even though off-target effects were most frequently analyzed with PCR followed by sequencing [81], mutations at further loci of the target sequences may be overlooked with the few sequenced clones [82]. Although whole-genome sequencing can remediate the limitation, it is associated with high costs [83]. In terms of in vivo delivery of CRISPR-Cas9, Charlesworth et al. showed some healthy human adults had preexisting anti-Cas IgG antibodies and anti-Cas9 T cells. These preexisting adaptive immune responses may lead to immune inactivation of Cas9 and further decrease the editing efficiency [84].

Functional nucleic acids, such as aptamers, have shown promise in various diagnostic and therapeutic applications since their discovery in 1990. Aptamer’s superior binding profile and molecular properties have allowed it to be integrated into the CRISPR/Cas system. The early research has shown that aptamers can aid in overcoming the off-target issue inherent in the CRISPR/Cas system. Furthermore, researchers have been able to harvest aptamers’ versatility toward a wide variety of targets and successfully developed many proof-of-concept diagnostics and biosensing assays. With the continued development of the CRISPR/Cas technology and the identification of new aptamers, it is reasonable to believe that the scientific community will see an increase in their integration. Like many other new areas of study, successful integration and field deployment of the novel technology will require interdisciplinary collaboration between biologists, chemists, and engineers. This is a unique opportunity that awaits every scientist in the near future.

## Data Availability

Not applicable.
